# Blockade of the Adenosine 2A Receptor Mitigates the Cardiomyopathy Induced by Loss of Plakophilin-2 Expression

**DOI:** 10.3389/fphys.2018.01750

**Published:** 2018-12-05

**Authors:** Marina Cerrone, Chantal J. M. van Opbergen, Kabir Malkani, Natasha Irrera, Mingliang Zhang, Toon A. B. Van Veen, Bruce Cronstein, Mario Delmar

**Affiliations:** ^1^Leon H. Charney Division of Cardiology, NYU School of Medicine, New York, NY, United States; ^2^Department of Medical Physiology, Division of Heart and Lungs, University Medical Center Utrecht, Utrecht, Netherlands; ^3^Division of Translational Medicine, NYU School of Medicine, New York, NY, United States; ^4^Department of Clinical and Experimental Medicine, University of Messina, Messina, Italy

**Keywords:** arrhythmogenic right ventricular cardiomyopathy, plakophilin-2, adenosine, fibrosis, desmosomes, cardiomyopathy, ATP, mouse models of arrhythmias

## Abstract

**Background:** Mutations in plakophilin-2 (PKP2) are the most common cause of familial Arrhythmogenic Right Ventricular Cardiomyopathy, a disease characterized by ventricular arrhythmias, sudden death, and progressive fibrofatty cardiomyopathy. The relation between loss of PKP2 expression and structural cardiomyopathy remains under study, though paracrine activation of pro-fibrotic intracellular signaling cascades is a likely event. Previous studies have indicated that ATP release into the intracellular space, and activation of adenosine receptors, can regulate fibrosis in various tissues. However, the role of this mechanism in the heart, and in the specific case of a PKP2-initiated cardiomyopathy, remains unexplored.

**Objectives:** To investigate the role of ATP/adenosine in the progression of a PKP2-associated cardiomyopathy.

**Methods:** HL1 cells were used to study PKP2- and Connexin43 (Cx43)-dependent ATP release. A cardiac-specific, tamoxifen-activated PKP2 knock-out murine model (PKP2cKO) was used to define the effect of adenosine receptor blockade on the progression of a PKP2-dependent cardiomyopathy.

**Results:** HL1 cells silenced for PKP2 showed increased ATP release compared to control. Knockout of Cx43 in the same cells blunted the effect. PKP2cKO transcriptomic data revealed overexpression of genes involved in adenosine-receptor cascades. Istradefylline (an adenosine 2A receptor blocker) tempered the progression of fibrosis and mechanical failure observed in PKP2cKO mice. In contrast, PSB115, a blocker of the 2B adenosine receptor, showed opposite effects.

**Conclusion:** Paracrine adenosine 2A receptor activation contributes to the progression of fibrosis and impaired cardiac function in animals deficient in PKP2. Given the limitations of the animal model, translation to the case of patients with PKP2 deficiency needs to be done with caution.

## Introduction

Arrhythmogenic right ventricular cardiomyopathy (ARVC, indicated also as “arrhythmogenic cardiomyopathy, ACM” or “arrhythmogenic right ventricular dysplasia, ARVD”) is an inherited disease characterized by fibrofatty infiltration of right ventricular (RV) predominance, ventricular arrhythmias and high propensity for sudden death in the young (Basso et al., 2009). Cardiac arrest is often associated with exercise, and it is the first disease manifestation in a high proportion of probands.(Basso et al., 2009; Groeneweg et al., 2015) Most often the disease begins with a subclinical, concealed stage, followed by overt stages of RV predominance (though LV is often involved) then bi-ventricular dilated cardiomyopathy and failure.(Basso et al., 2009; Groeneweg et al., 2015) The disease can progress to end-stage heart failure and in the absence of a heart transplant, death. At present, no medical treatment exists to prevent or delay the progression of the disease.

Familial ARVC is most commonly consequent to mutations in the gene coding for Plakophilin-2 (PKP2; Groeneweg et al., 2015), a protein classically defined as a component of the desmosome, though also known to participate in other cellular functions. The relation between PKP2 on one hand, and the structural disease that results from its absence or altered sequence, on the other, remains a matter of investigation. Previous studies on the molecular mechanisms leading to fibro-adiposis in ARVC have proposed that activation of the Hippo and Wnt pathways are the causative mechanisms of the structural disease (Garcia-Gras et al., 2006; Chen et al., 2014). Recently, Dubash et al. (2016) demonstrated an additional route whereby loss of PKP2 expression causes an increase in the expression of TGF-beta1, and activation of the p38-MAPK-dependent pro-fibrotic program. Importantly, and in contrast with previous studies that have characterized the activation of profibrotic genes as cell-autonomous processes, Dubash et al. (2016) showed that p38-MAPK was also activated in PKP2-expressing cells neighboring those lacking PKP2 expression. These data strongly suggested the presence of a paracrine pathway for induction of the pro-fibrotic program. The molecular carrier of this cell-cell message and the additional possibility of autocrine regulation were not characterized in that study.

There are a number of potential molecular carriers of cell-cell information. In the present study, we have focused on adenosine. Studies from the Cronstein laboratory have shown that binding of adenosine to the adenosine 2A receptor (A2AR), a member of the large family of G protein coupled receptors, significantly enhances collagen deposition in skin, lung and liver tissues by indirect mechanisms that include stimulation of growth factor expression (TGF-beta and CTGF) as well as by direct stimulation of matrix production (Shaikh and Cronstein, 2016). Recent observations from the same lab have further pointed to cross-talk between A2AR activation and the Wnt-signaling pathway in promoting matrix production (Shaikh et al., 2016; Zhang et al., 2017). Separate studies from others (Jeon et al., 2011; Giambelluca et al., 2013) have also shown that A2AR stimulation activates GSK3-beta, a signaling molecule in the Wnt pathway that is potentially involved in ARVC (Asimaki et al., 2014). The cumulative data indirectly suggest a possible role for adenosine as a mediator of the ARVC phenotype; yet, the latter remains to be assessed.

Increased levels of adenine nucleotides in the extracellular space give rise to adenosine in models of both dermal and hepatic fibrosis as a result of nucleoside triphosphate dephosphorylase (CD39)- and ecto 5′-nucleotidase (CD73)-mediated dephosphorylation of adenine nucleotides (Peng et al., 2008; Fernandez et al., 2013). Importantly, studies in a number of systems have indicated that (a) connexin43 (Cx43) hemichannels allow for the release of ATP (Bol et al., 2017), and (b) there is a physical association between Cx43 and the desmosome, in a protein complex referred to as the connexome (Agullo-Pascual and Delmar, 2012). We thus speculated that PKP2 deficiency may facilitate opening of Cx43 hemichannels and favor ATP release. We also speculated that blockade of the A2AR in the heart may slow progression of the cardiomyopathy that results from loss of PKP2 expression (Cerrone et al., 2017). These two hypotheses were tested in the present study.

## Materials and Methods

### Generation of CX43 Deficient HL1 Cell Line

HL-1 is a cardiac muscle cell line derived from the AT-1 mouse atrial cardiomyocyte tumor lineage. To generate a stable CX43-deficient cell line (CX43-KD) we utilized a lenti-GJA1-shRNA clone (Clone ID TRCN0000068473), containing a hairpin sequence targeting the 3′-UTR of GJA1 gene CCGGCCCACCTTTGTGTCTTCCATACTCGAGTATGGAAGACACAAAGGTGGGTTTTTG. A separate line was generated that expressed a non-silencing Lenti vector (CX43 ΦKD) and used as a control. Both control and CX43-KD HL-1 cells were selected in Claycomb medium containing puromycin. The silencing of CX43 expression was confirmed by Western Blot. Briefly, the cells were first cultured in 6 well plate to 80% confluent. Cells were collected in PBS and pelleted by centrifugation at 2000 rpm 5 min at 4°C. The cell pellets were homogenized in lysis buffer (150 mM NaCl, 0.02% Sodium azide, 1% Triton X-100, 1 mM PMSF, 1 mM Na_3_VO_4_, 50 mM NaF, 50 mM Tris–HCl, pH 8.0 and Complete Protease Inhibitor) by vortex on ice for 3 times (10 s each). The supernatant were obtained by centrifugation at 8,000 rpm at 4°C for 5 min. Protein concentration was determined using BCA Kit (Thermo Fisher). Samples were run on 4–15% precast polyacrylamide SDS gradient gels (Bio-Rad) and transferred onto nitrocellulose membranes and subsequently incubated in blocking buffer consisting of PBS with Tween-20 (0.1%) and 1% non-fat dry milk. Membrane was then incubated with CX43 (Millipore) and PKP2 (Fitzgerald International) antibodies diluted in 1% non-fat dry milk T-PBS overnight at 4°C. The secondary antibodies goat-anti-rabbit IRDye 800CM and goat anti-mouse IRDye 680RD were followed after wash in T-PBS buffer. The image was obtained by scanning with an Odyssey Fc Imaging System (LI-COR).

### ATP-Release Experiments in PKP2 Deficient and PKP2/Cx43 Deficient HL1 Cell Line

The ATP release assay was performed with HL-1 control (CTL), PKP2 deficient [PKP2-KD (Cerrone et al., 2014)] and PKP2/CX43 double deficient (PKP2/CX43-KD) HL-1 cells. Briefly, cells were treated with hypotonic solution (1.2 mM CaCl_2_, 1.8 mM MgCl_2_, 25 mM HEPES pH 7.4) at 37°C for 30 min. The medium supernatant was collected from each cell well and mixed with the reaction solution. The released ATP was determined using a bioluminescence assay in which ATP-dependent generation of light by recombinant firefly luciferase and its substrate D-luciferin was measured with an ATP Determination Kit (Feig et al., 2017) (Life Technologies). The relative luminescence was detected by plate reader Flex 3 Station. All assays were performed in triplicate. Data were from at least 4 independent experiments and normalized to HL-1 control cells.

### Mouse Model

The PKP2cKO animal model used in this work was the C57BL/6 PKP2 fl/fl mice line generated and crossed with the αMyHC-Cre-ER(T2) line described for the first time in Cerrone et al. (2017). Details on the generation and characterization of the αMHC-Cre-ER(T2) line can be found in Takefuji et al. (2012). Regarding the PKP2cKO line, 2 forward loxP sites were designed and introduced into the construct flanking mouse PKP2 exons 2 and 3, with a downstream neomycin selection cassette. The linearized targeting construct was electroporated into C57BL/6 derived embryonic stem cells and the resulting embryonic stem cell clones were identified. The confirmed positive embryonic stem cells were injected into isogenic blastocysts and microinjected into the foster mice. The neo cassette was excised by crossing F1 heterozygous with FRT mice. The PKP2 fl/fl mice were mated to αMyHC-Cre-ER(T2) (C57BL/6 background; the αMyHC-Cre-ER(T2) construct has been passed into C57Bl/6 mice for more than 15 generations) mice in order to obtain flox/flox/Cre+ mice which contain the αMyHC promoter and the ligand binding domain of the human estrogen receptor. The mice resulting from the crossing develop normally.

Mice were injected 4 consecutive days with tamoxifen. Binding of tamoxifen to the estrogen receptor induced the cardiomyocyte specific Cre-mediated deletion of the *Pkp2* gene. All experiments were performed in PKP2-cKO mice and Cre-negative, tamoxifen treated, littermate were used as controls for transcriptome experiments. Untreated PKP2cKO mice were used as controls for the pharmacological interventions experiments.

Considering that the initial characterization of this mouse model (Cerrone et al., 2017) did not show phenotype differences between genders, animals of both genders and between 3 and 4 months old were used for the experiments.

All procedures conformed to the Guide for Care and Use of Laboratory Animals published by the United States National Institutes of Health (NIH Publication no. 58-23, revised 1996) and were approved by the NYU IACUC (protocol #160726-01).

### Pharmacological Interventions

PKP2cKO were injected with istradefylline [ISTRA, Sigma Aldrich, 5–10 mg/Kg/day i.p.(Zhang et al., 2017)] or with PSB115 [TOCRIS, 15 mg/Kg/day i.p.(Hayallah et al., 2002; Abo-Salem et al., 2004)] from 14 to 35 days post tamoxifen injection (dpi).

### Echocardiography

Transthoracic echocardiography was performed using a Vevo2100 Imaging System (VisualSonics Inc., Toronto, Canada) with a 30 MHz probe. Briefly, after induction of anesthesia in a chamber containing isoflurane 4–5% in oxygen, the mouse was positioned supine on a heat pad in order to maintain body temperature at 37–38°C and anesthesia was maintained with 1.5% isoflurane in 700 ml O_2_/minute via a nose-cone. Recordings were obtained in parasternal long and short axis views (Cerrone et al., 2017). Quantitative measurements were assessed offline using the Vevo2100 analytical software. A B-mode parasternal long axis view was used for left ventricular ejection fraction measures.

### Histology

Hearts were fixed with 4% paraformaldehyde in phosphate buffered saline (PBS), embedded in paraffin, and cut into 5 μm thick sections. Sections were stained with Masson’s Trichrome according to the manufacturer’s instructions. Stained sections were scanned at a 40X magnification on a Leica SCN400F Whole Slide Scanner. The ImageJ (NIH) software was used for analysis of tissue section, as in Cerrone et al. (2017). By defining regions of interest (ROIs), three ROIs for each ventricle were selected (base, free mid-wall, apex) and the interventricular septum was excluded. For each ROI, the area of collagen (blue staining) was normalized to the area of tissue.

### Picrosirus Red and Immunohistochemistry

Both picrosirius red and immunofluorescence were performed on thin sections from paraffin embedded hearts. For immunofluorescence, tissue sections were deparaffinized and rehydrated; antigen retrieval was performed for 15 min at 37°C with proteinase K solution (20 μg/ml in Tris EDTA buffer, pH 8.0). Thin sections from both free ventricular walls were incubated with PBS containing 5% Fetal Bovine Serum, 3% Bovine Serum Albumin (BSA) and Triton X-100 1% (1 h at RT). Samples were incubated with primary antibodies [mouse monoclonal anti-vimentin (1:200)- Santa Cruz Biotechnology, CA, United States] overnight at 4°C in a humidified chamber. The day after, samples were washed with 3% PBS-BSA and incubated with secondary antibodies [anti-mouse IgG-Alexa Fluor 555 (red)] for 1 h. Nuclei were counterstained with DAPI mounting medium. Immunofluorescence images were collected with an Eclipse NI-U fluorescence upright microscope (Nikon). For the measurement of fluorescence intensity, all images were captured at the same exposure times, contrast settings, and intensity. Picrosirius red polarized images were collected with a Nikon DS-Fi1 camera (Tokyo, Japan), green and red fibers from left and right ventricles were quantified with Image J software.

### RNAseq and KEGG Analysis

Data mentioned in this study were derived from the initial set generated in Cerrone et al. (2017).

### Statistical Analysis

All data are expressed as mean ± SEM. Comparisons between experimental groups were analyzed by Student *T*-test or one-way ANOVA for non-parametric variables with Tukey’s post-test for intergroup comparisons (SPSS v.24, IBM). A *p* value of < 0.05 was considered statistically significant. Graphs were drawn using GraphPad Prism (version 5.0 for Windows).

## Results

### ATP Release in PKP2-Deficient HL-1 Cells

**FIGURE 1:**
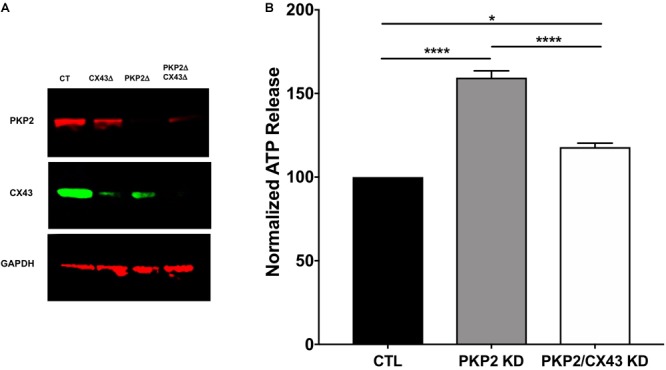
**(A)** Western blot confirming knock-down of expected proteins in CX43Δ, PKP2Δ, and PKP2Δ/ CX43Δ HL-1 cell lines. **(B)** ATP release measured in HL1 cells (black bar), PKP2-deficient HL1 cells (PKP2KD, gray bar) and PKP2 and connexin43-deficient HL1 cells (PKP2/CX43 KD, white bar). ATP levels are higher in PKP2KD cells when compared to controls. Concomitant loss of Cx43 blunts partially this effect. ^∗^*p* < 0.05; ^∗∗∗∗^*p* < 0.0001.

We examined the relation between PKP2 expression and ATP release using HL1 cells stably expressing a PKP2 silencing RNA [PKP2-KD; see (Cerrone et al., 2014)] compared to cells expressing a scrambled sequence (control, or “ctrl”). Details in the design and characterization of this line have been described before (Cerrone et al., 2014) and are consistent with methods used by other investigators (Chen et al., 2014). ATP release was provoked by a shift in osmotic pressure, as previously described (van der Wijk et al., 2003). As shown in Figure 1, the release of ATP was significantly larger in cells deficient in PKP2, suggesting that reduced expression of the desmosomal protein led to the opening of a hydrophilic pathway for ATP release from the intracellular space, likely Cx43 hemichannels. We therefore examined ATP release in a cell line where both PKP2 and Cx43 were silenced (line PKP2/Cx43-KD; see methods and Figure 1 for details). Loss of Cx43 expression drastically blunted the release of ATP (Figure 1). These results suggest that loss of PKP2 expression is linked to Cx43-mediated release of ATP to the extracellular milieu.

### Downstream Targets of A2AR Activation Are Upregulated in PKP2cKO Mice

**FIGURE 2:**
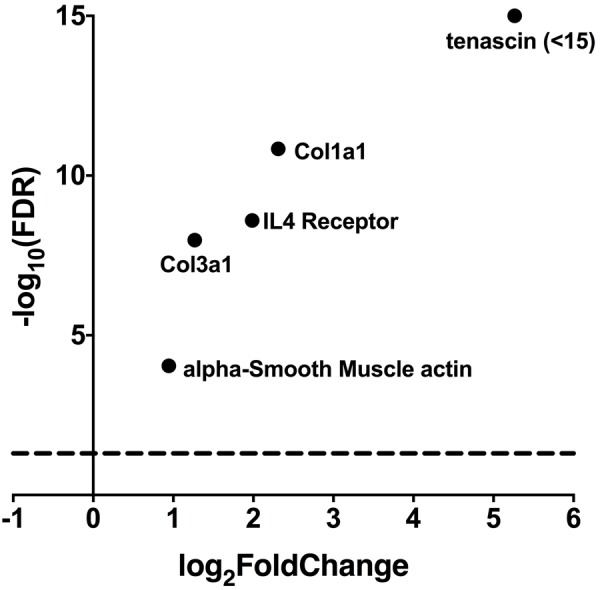
RNAseq data from PKP2cKO (*n* = 5) and littermate control (*n* = 4) mice showing that lack of PKP2 expression is linked to overexpression of genes involved in fibrosis and regulated by adenosine expression (Perez-Aso et al., 2014; Ferrari et al., 2016). In the abscissae, “log_2_FoldChange” refers to the magnitude of the differential (expressed as logarithm base 2) of the abundance of a given transcript in control when compared to that present in PKP2cKO mice hearts. The ordinates indicate the negative logarithm base 10 of the confidence (*p*-value) calculated from the difference between abundance in control versus abundance in the PKP2cKO.

In the heart as in other tissues, ATP is rapidly converted to adenosine, a well-known agonist for receptor-activated intracellular signaling (Borea et al., 2016). We therefore examined whether transcript levels for genes downstream of the adenosine receptors, were upregulated in hearts deficient in PKP2. For this purpose, we examined the differential transcriptome of a cardiac-specific, tamoxifen activated PKP2 knockout murine line (PKP2cKO) previously described by our laboratory (Cerrone et al., 2017). Trascriptome data are available as Supplementary Table S1. Figure 2 shows how transcripts known to be part of adenosine receptor-initiated cascades (Perez-Aso et al., 2014; Ferrari et al., 2016) were over-represented in the PKP2cKO hearts in respect to the controls.

### Istradefylline, an A2AR Blocker, Prolongs Survival and Partially Preserves LVEF in PKP2cKO Mice

**FIGURE 3:**
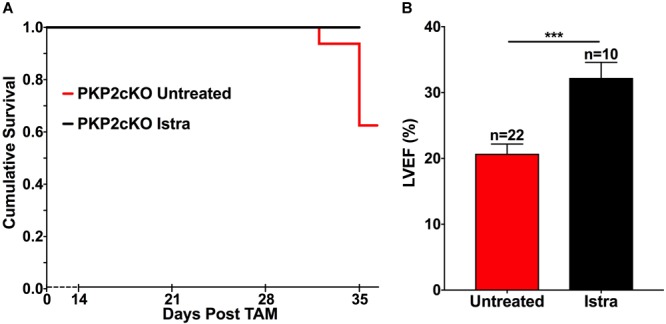
**(A)** Kaplan Meyer survival curve showing that PKP2cKO mice treated with ISTRA (black line, *n* = 10) have increased survival when compared to untreated ones (red line, *n* = 22). **(B)** Left Ventricular ejection fraction (LVEF) in PKP2cKO mice 35 days after tamoxifen injection. ISTRA-treated mice (black bar, *n* = 10) showed improved values when compared to untreated mice (red bars, *n* = 22). ^∗∗∗^*p* < 0.001.

The results noted above suggested that adenosine acts as a paracrine agonist in the setting of PKP2 deficiency. We therefore examined whether an A2AR blocker could change the course of the cardiomyopathy that results from PKP2 deficiency. For this purpose, mice were treated with 5–10 mg/Kg/day of ISTRA (Dungo and Deeks, 2013; Zhang et al., 2017), delivered by intraperitoneal injection and starting 14 days post-tamoxifen injection (14 dpi), that is, at a time prior to the onset of the cardiomyopathic phenotype (Cerrone et al., 2017). As shown in Figures 3A,B and as previously reported (Cerrone et al., 2017), the mortality rate at 35 dpi in PKP2cKO deficient mice was 37.5%, and LVEF in surviving animals was markedly reduced (21% +/- 1.46%; *n* = 22). In the presence of ISTRA, no deaths were observed (Figure 3A) and LVEF was partially preserved (32% +/- 2.35%; *n* = 11; *p* < 0.001; Figure 3B). These results indicate that pharmacologic blockade of the A2AR can prolong life expectancy and partially interrupt the progression of the cardiomyopathic phenotype.

### ISTRA Effect on Fibrosis in PKP2cKO Mice

**FIGURE 4:**
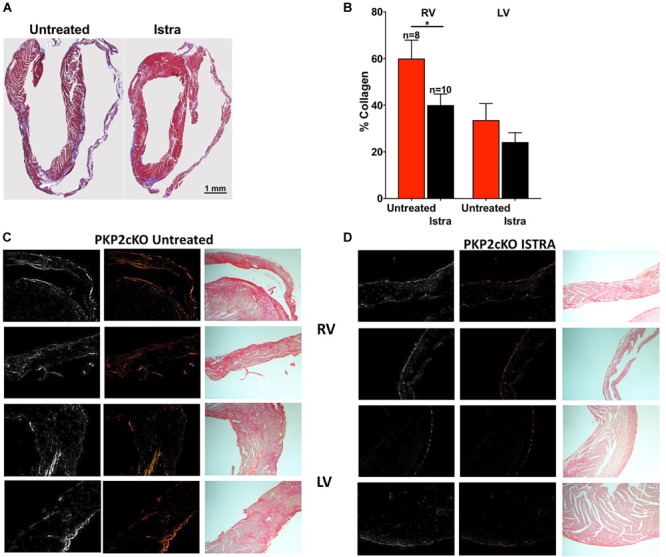
**(A)** Masson Trichrome staining of untreated (left) and ISTRA-treated (right) PKP2cKO mice hearts 35 days post-tamoxifen injection. **(B)** Collagen percentage in the right (left bars) and left (right bars) ventricles of PKP2cKO mice treated (black, *n* = 10) and untreated (red, *n* = 8) with ISTRA. ^∗^*p* < 0.05. **(C,D)** Picrosirius red staining in areas of the right (top) and left (bottom) ventricles in PKP2cKO untreated and ISTRA-treated hearts. Left panels show BW contrast and polarized light microscopy images.

**FIGURE 5:**
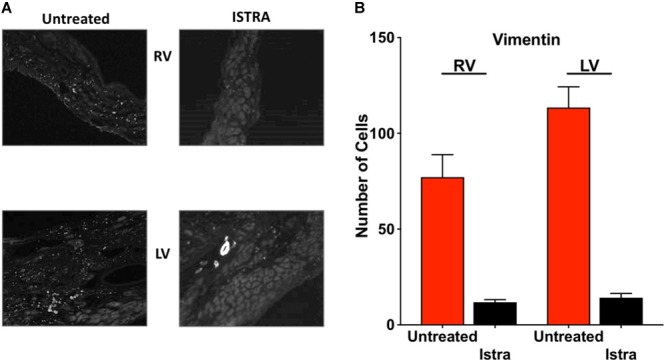
**(A)** Examples of vimentin staining on sections of PKP2cKO ventricles at 35 days post tamoxifen untreated (left) and after ISTRA treatment (right), showing decreased fibroblast abundance. Note the vessel in the right bottom panel (LV) as positive control for appropriate staining. **(B)** Quantification of vimentin-positive cells in untreated (red bar) and ISTRA treated hearts (black bars).

Given the known pro-fibrotic role of A2AR activation, we examined whether ISTRA changed the course of fibrosis that occurs after loss of PKP2 expression (Cerrone et al., 2017). As shown in Figures 4A,B, we observed reduced collagen abundance in the right ventricular free wall in ISTRA-treated mice. A similar though not significant tendency was observed in the left ventricular free wall. In more detailed analysis we observed areas void of collagen staining in both free walls of PKP2cKO hearts compared to controls (Figures 4C,D). Moreover, we found a drastic reduction in the abundance of vimentin-positive cells in the ISTRA-treated hearts compared to the untreated controls (Figures 5A,B), suggesting that the A2AR antagonist limits the recruitment of non-myocyte cells into the heart interstitial space after loss of PKP2 at the time tested.

### A2B Receptor Blockade Is Pro-fibrotic in PKP2cKO Hearts

**FIGURE 6:**
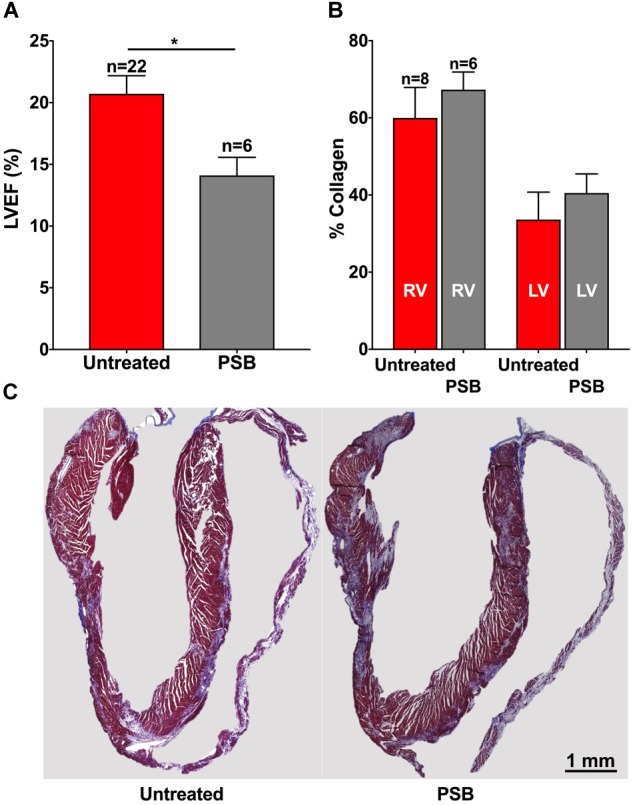
**(A)** Left ventricular ejection fraction is worsened by PSB115 treatment in PKP2cKO mice 35 days after tamoxifen injection. **(B)** Treatment with PSB115 does not reduce fibrosis in the right (RV) or left ventricles (LV) in PKP2cKO mice and shows a trend toward increased fibrosis. **(C)** Masson trichrome stained examples of untreated (left) and PSB115-treated (right) hearts of PKP2cKO mice at 35 days post tamoxifen injection. Red bars: untreated animals. Gray bars: treated with PSB115. ^∗^*p* < 0.05.

ISTRA is an A2AR-selective antagonist. To further confirm the specificity of A2AR involvement in cardiac fibrosis in these mice we tested the effects of PSB115, a selective antagonist of A2BAR (Hayallah et al., 2002; Abo-Salem et al., 2004). Interestingly, the results were opposite to those observed with ISTRA. Hearts treated with PSB115 (15 mg/Kg/day treated between 14 and 35 dpi) showed impaired LVEF (14% *p* < 0.01 vs. untreated, Figure 6A) and a trend toward increased fibrosis (Figures 6B,C) when compared to control, thus suggesting that A2A but not A2B receptors can be considered a potential target for treatment in PKP2-dependent cardiomyopathies.

## Discussion

Plakophilin-2 was originally described as a desmosomal molecule. Evidence over the last 10 years has shown that, in addition to its role in cell-cell adhesion, PKP2 scaffolds an intracellular signaling hub at the cell junction, and also participates in the control of transcription. The involvement of the same molecule in multiple functions may explain, at least in part, the complex phenotype that results from its deficiency, ranging from electrical diseases such as CPVT (Anderson et al., 2016) to electrical/structural [Brugada syndrome (Cerrone et al., 2014)] to mainly structural [biventricular dilated cardiomyopathy and end-stage failure (Groeneweg et al., 2015)]. Given the pleiotropism of PKP2, it is unlikely that a “masterswitch” will be found downstream of PKP2 that can prevent all phenotypical features. Yet, analysis of the relation between PKP2 and specific interacting molecules has been helpful in identifying molecular cascades and events that explain at least some aspect of the overall phenotype. Here, we concentrate on one possible mechanism for the paracrine effect resulting from PKP2 loss, namely, the extrusion of ATP from PKP2-deficient cells and the activation of adenosine receptors.

The availability of the PKP2cKO murine model has allowed us to examine whether drugs that interfere with pro-fibrotic cascades can blunt the progression of a cardiomyopathy caused by PKP2 deficiency. Our approach has been not to design new drugs or examine compounds only for experimental use but rather, to test whether existing approved drugs can be re-purposed for the specific case of ARVC [e.g., flecainide to treat triggered activity-initiated arrhythmias; see (Cerrone et al., 2017)]. Drug repurposing -i.e., the identification of new uses for existing drugs- is a highly efficient bypass to the expensive and time-consuming process of drug development. Here we examined istradefylline (Hickey and Stacy, 2012; Dungo and Deeks, 2013; Muller, 2015) (ISTRA; first coded KW6002), a selective A2AR antagonist. Our data show that ISTRA partially preserves left ventricular function in the setting of PKP2 deficiency.

ISTRA is a highly selective antagonist of the adenosine 2A receptor (Hickey and Stacy, 2012; Dungo and Deeks, 2013). The drug is currently in use in Japan as adjunctive therapy in Parkinson disease and studies over long periods demonstrate that the daily oral administration of the drug is well tolerated and safe (Muller, 2015). No use of ISTRA in the context of heart disease has been reported to date. Yet, evidence in the literature (reviewed in the introduction) suggested, albeit indirectly, a potential for convergence of A2AR-mediated signaling pathways with those that participate in the PKP2-dependent cardiomyopathic phenotype. This molecular intersection was further explored in the present study. Our data show that even if not completely effective, mice treated with ISTRA showed a partial preservation of LVEF 5 weeks after the loss of PKP2 expression. Though it is far too soon for speculation in the clinical realm, it is worth noting that, if ISTRA were to have a similar effect in human ARVC, this effect could be beneficial to those patients progressing toward end-stage heart failure with the only prospect of either heart transplant or death.

We examined the relation between PKP2 expression and ATP release in HL1 cells deficient in PKP2 (PKP2-KD)(Cerrone et al., 2014). HL1 cells have been used before both by us and by other investigators to study PKP2 function and shown to yield results consistent with those observed in cardiac preparations (Cerrone et al., 2014; Dubash et al., 2016). The increased abundance of ATP in the extracellular milieu of stimulated PKP2-KD cells suggested the formation of an ATP-permeable hydrophilic pathway in the cells after PKP2 loss. Given the association between molecules of mechanical and of electrical junctions into a common protein interacting network [the connexome (Agullo-Pascual and Delmar, 2012)], we speculated that Cx43 hemichannels may mediate ATP release. The latter was supported by experiments demonstrating that loss of Cx43 expression blunted the PKP2-dependent release of ATP. It is tempting to speculate that the reduced intercellular adhesion strength consequent to loss of PKP2 expression creates a pool of Cx43 connexons that would otherwise form gap junctions. These hemichannels can passively allow leakage of intracellular solutes, including ATP. In that regard, it is worth noting that mitochondria abundantly localize to the site of cell-cell contact and as such, the intercellular junctions are expected to be rich on ATP (Leo-Macias et al., 2015).

In contrast to the effect of A2AR blockade, we found that blockade of A2B receptors worsened fibrosis in the PKP2 deficient mice. Adenosine A2B receptors have been reported to play divergent roles in cardiac fibrosis; in some models A2B receptors inhibit and in other models promote fibrosis [Reviewed in Vecchio et al. (2017)]. A number of *in vitro* studies demonstrate that A2B receptor stimulation inhibits cardiac fibroblast expression of pro-fibrotic genes and their products and in *in vivo* studies A2B receptor stimulation blocks cardiac fibrosis induced by adenosine infusions. In contrast, in models of acute myocardial infarction or other injury models A2B receptor blockade inhibits cardiac fibrosis. The results of the studies reported here are most consistent with models in which acute cardiac injury leads to cardiac fibrosis and adenosine A2B receptor stimulation prevents fibrosis since A2B receptor blockade worsened the cardiac fibrosis observed in the PKP2cKO mice. Although A2A and A2B adenosine receptors share signal transduction through G_aS,_ adenylate cyclase and protein kinase A or EPAC1/2, A2B receptors also signal through G_q_ (Fredholm et al., 2011), thus it is possible that biasing the signal transduction of A2B receptors toward one path or the other would lead to very different outcomes and A2B blockade could have the same or opposing effect on cardiac fibrosis as A2AR blockade.

Although long-term increases in adenosine levels in the heart may promote cardiac fibrosis, as we have observed, it has long been reported that adenosine release is increased from the heart after exercise and during ischemia (Bardenheuer et al., 1994) and has beneficial effects. Adenosine A2AR mediate suppression of cardiac injury in animal models of myocardial infarction (Lozza et al., 1997) and beneficial effects of adenosine infusion have been noted in patients after myocardial infarction (Garratt et al., 1998) as well. Nonetheless, it is tempting to speculate that these acute changes in adenosine levels following exercise may form the basis for exercise-induced worsening of cardiac prognosis in patients with mutations in *Pkp2*.

In summary, our results indicate that A2AR activation contributes, likely via the release of ATP by Cx43 hemichannels, to the myriad of intracellular signaling cascades that initiate and crisscross after loss or mutations in PKP2. In that regard, our data support the notion that a pharmacological intervention on this specific pathway may ameliorate the structural damage caused by desmosomal dysfunction. Yet, given the fundamental differences between experimental models and clinical conditions, applicability of our data to the case of the patient with ARVC remains purely speculative.

## Data and Materials Availability

The datasets used and/or analyzed during the current study are available from the corresponding author on reasonable request.

## Author Contributions

MC designed the study, coordinated the study, acquired the data, analyzed the data, interpreted the data, and prepared the manuscript. CvO acquired the data, analyzed the data, and analyzed the statistical. KM acquired the data, analyzed the data, analyzed the statistics, and prepared the figures. NI acquired and analyzed the data and prepared the figures. MZ acquired the data and prepared the figures. TVV critically revised and approved the manuscript. BC designed the study, prepared the manuscript, critically revised the manuscript, secured the funding, and approved the manuscript. MD conceived and designed the study, secured the funding, prepared the manuscript, and critically revised the data.

## Conflict of Interest Statement

The authors declare that the research was conducted in the absence of any commercial or financial relationships that could be construed as a potential conflict of interest.

## Supplementary Material

TABLE S1 RNA-Seq data of differentially expressed genes in PKP2-CKO and control hearts at 21 days post tamoxifen injection.
